# Contact-dependent delivery of IL-2 by dendritic cells to CD4 T cells in the contraction phase promotes their long-term survival

**DOI:** 10.1007/s13238-019-00662-0

**Published:** 2019-11-05

**Authors:** Dan Tong, Li Zhang, Fei Ning, Ying Xu, Xiaoyu Hu, Yan Shi

**Affiliations:** 1 Tsinghua Institute for Immunology and Department of Basic Medical Sciences, Beijing Key Lab for Immunological Research on Chronic Diseases, School of Medicine, Tsinghua-Peking Center for Life Sciences, Tsinghua University, Beijing, 100084 China; 2 Department of Microbiology, Immunology and Infectious Diseases, Snyder Institute, University of Calgary, Calgary, Canada

**Keywords:** dendritic cell, contact dependence, long term survival, T cell memory, IL-2

## Abstract

Common γ chain cytokines are important for immune memory formation. Among them, the role of IL-2 remains to be fully explored. It has been suggested that this cytokine is critically needed in the late phase of primary CD4 T cell activation. Lack of IL-2 at this stage sets for a diminished recall response in subsequent challenges. However, as IL-2 peak production is over at this point, the source and the exact mechanism that promotes its production remain elusive. We report here that resting, previously antigen-stimulated CD4 T cells maintain a minimalist response to dendritic cells after their peak activation *in vitro*. This subtle activation event may be induced by DCs without overt presence of antigen and appears to be stronger if IL-2 comes from the same dendritic cells. This encounter reactivates a miniature IL-2 production and leads a gene expression profile change in these previously activated CD4 T cells. The CD4 T cells so experienced show enhanced reactivation intensity upon secondary challenges later on. Although mostly relying on *in vitro* evidence, our work may implicate a subtle programing for CD4 T cell survival after primary activation *in vivo*.

**Electronic supplementary material:**

The online version of this article (10.1007/s13238-019-00662-0) contains supplementary material, which is available to authorized users.

## Introduction

In the linear model of T cell memory (Gasper et al., 2014), long lasting memory cells develop out of the contracting phase of activation. Cytokines, particularly IL-7 and IL-15, have been implicated in the formation, homeostasis and subtype differentiation of memory T cells (Gasper et al., 2014; Raeber et al., 2018). With all these advances in our understanding, exact determinant that leads to T cells’ commitment to memory is not clearly understood, particularly with regard to CD4 T cells (Bell and Westermann, 2008; MacLeod et al., 2009). Despite of different proposed mechanisms, a common question remains whether cell extrinsic factors can guide their long-term survival following primary infection.

Compared with IL-7 and IL-15, IL-2 signaling in memory T cell formation is less understood. For CD8^+^ T cells, IL-2 in the primary infection expands the cell number and renders them resistant to secondary challenges, mainly via anti-apoptotic signaling (Liao et al., 2013). In 2006 Bevan’s group reported that to establish CD8 T cell memory, the presence of CD25 on these cells during primary infection is critical. In that report, the authors ruled out autocrine IL-2, instead suggested an environmental source, such as CD4 T cells or dendritic cells (DCs) (Williams et al., 2006). This signaling was regarded as a strong one as it could be replaced only with IL-2/IL-2 antibody complex. A later report, however, suggested that CD8^+^ T cells could produce IL-2 for their own use in memory formation (Feau et al., 2011). Interestingly, it is generally agreed that a strong and sustained IL-2 signaling via CD25 leads to a terminal effector differentiation, detrimental to memory development (Kalia et al., 2010). While these findings may not be mutually exclusive, it does however, seem to indicate a discreet and managed availability of IL-2 at this stage of T cell activation. A similar analysis was performed for the role of IL-2 in CD4 T cell memory as well (McKinstry et al., 2014). IL-2 was found to be critical for the expression ratio of Blimp vs. Bcl-2, promoting cell survival and at the same time upregulate CD127 (IL-7Rα). Again, the intensity of IL-2 signaling at this stage was considered to be high. Because in the absence of autocrine IL-2 (IL-2 deficient T cells), the memory development required high levels of external IL-2 or in the form of IL-2/IL-2 antibody complex, and was minimally rescued by co-transfer of IL-2 sufficient primed CD4 T cells (McKinstry et al., 2014). However, it is not clear why IL-2 is belatedly needed in the primary stimulation, and more ominously whether CD4 T cells produce sufficient IL-2 this late to support their own transition to memory (Sojka et al., 2004).

In this short report, we describe an *in vitro* subtle stimulation event in activated T cells late in the primary phase of activation. In an experiment to study the CD69 expression profile in a typical *in vivo* T cell activation event, we noticed the re-expression of CD69 on activated CD4 T cells several days after its peak expression *in vivo*. *In vitro*, a similar activation can be driven by DCs in an antigen independent manner. In our settings, contact of previously activated/rested CD4 T cells with dendritic cells (DCs) sent a very mild activation signal to these T cells and led to a re-production of IL-2. T cells after this mild stimulation, upon transfer into naïve recipients, can better maintain their response to antigen in a secondary stimulation. Instead of signaling via JAK/STAT pathways (Ross and Cantrell, 2018), RNA-seq analysis indicated this activation event enhanced the expression of *Bcl2* as well as *Oasl2* and *Isg15*, two type I interferon-related genes (Zhao et al., 2013; Choi et al., 2015). These results provide suggestive evidence that optimal secondary response requires a brief encounter with DCs following antigen clearance and imply that DCs could be the source of the “trickling” IL-2 in the later phase of T cell activation, potentially pointing to a missing link in the overall picture of CD4 T cell survival after their primary activation.

## Results

### Previously activated CD4 T cells upregulate CD69 in response to DC binding

**Figure 1:**
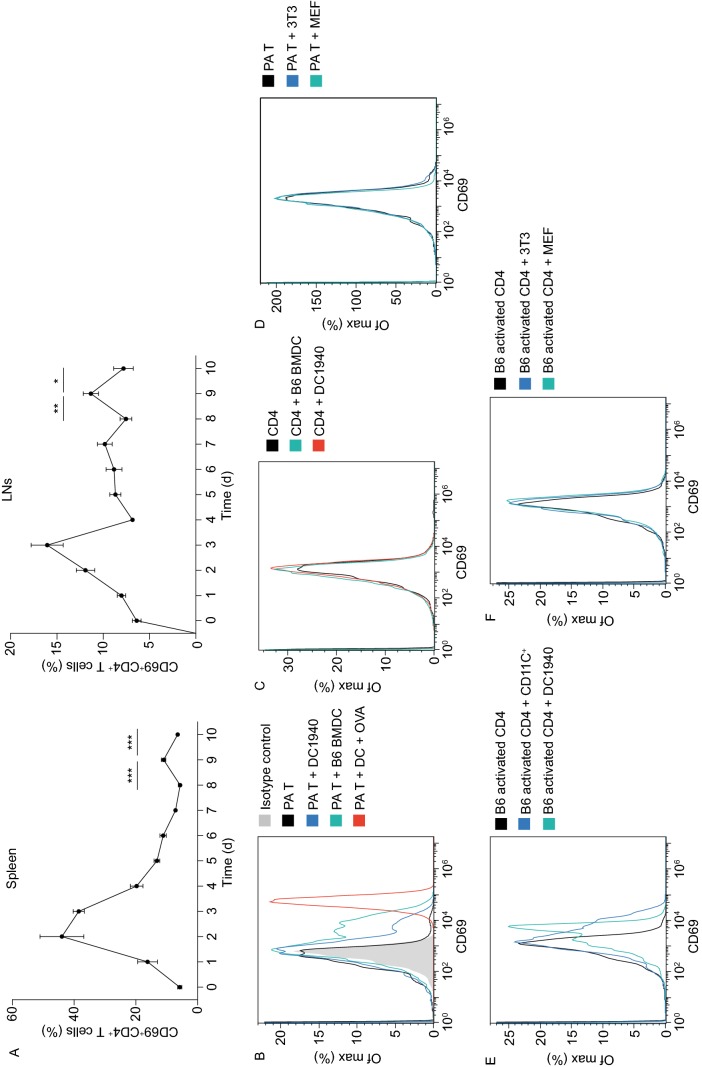
**PA T cells upregulate CD69 in DC co-culture**. (A) OT-II mice were i.v. injected with 0.1LD_50_ LM-OVA. dLNs (draining LNs) and spleen were harvested on stated days and CD69 expression on CD4 T cells as a percentage was determined by FACS. *n* = 5 mice per group, and total 55 mice in this experiment. Results are representative of three independent experiments (*N* = 3). *N* = 3 for independent repeats of the experiment. *P < 0.05, **P < 0.01, ***P < 0.001 (Unpaired Student’s *t* test). *n* (replicates of biological samples) and *N* (number of independent repeats of the experiments) designations, as well as statistical symbols are used henceforth. (B) Left: Representative staining of previously activated CD4 T cells (PA T) after resting 48 h, CD69 expression was compared with co-cultured with DC1940 cell-line or B6 BMDCs. Red line is positive control which stands for PA T co-cultured with DC1940 in the presence of 10 µg/mL OVA. Three replicates in each group (*n* = 3), results are representative of eight independent experiments (*N* = 8). Right: Pooled data from eight independent experiments are shown. Normalized CD69 mean fluorescence intensity (MFI) by the PA T group in multiple independently repeated experiments (*N* = 8) was analyzed for fold change of CD69 MFI. **P < 0.01, ****P < 0.0001 (Unpaired Student’s *t* test). (C) Similar to (B) except that naïve freshly magnetically isolated OT-II splenic CD4 cells were used in place of PA T. Three replicates in each group (*n* = 3), results are representative of three independent experiments (*N* = 3). (D) Similar to (B) except that B6 MEF and 3T3 cells were used in place of DCs. Three replicates in each group (*n* = 3), results are representative of four independent experiments (*N* = 4). (E) Magnetically isolated naïve CD4 T cells from B6 mice were activated *in vitro* with anti-CD3e and anti-CD28. Same experiment as in (B) was performed using B6 splenic CD11c^+^ cells and DC1940 as the stimulator. Three replicates in each group (*n* = 3), and results are representative of three independent experiments (*N* = 3). (F) Left: Similar to (D) except that MEF and 3T3 were in place of DCs. Three replicates in each group (*n* = 3), results are representative of three independent experiments (*N* = 3). Right: pooled data from three independent experiments are shown. Normalized CD69 mean fluorescence intensity (MFI) by the B6 activated CD4 group in multiple independently repeated experiments (*N* = 3) was analyzed for fold change of CD69 MFI. ns means no significant difference (Unpaired Student’s *t* test). Pooled data for the panels (C and D) from multiple experiments are shown in Fig. S3 as marked

In our previous work studying the effect of DC/T cell contact, we made an interesting finding. In OT-II mice challenged with *Listeria monocytogenes* expressing OVA (LM-OVA), the activated T cells isolated from the mice showed a typical rise in the percentage expression of CD69 in the primary response, followed by a gradual tapering off (Fig. 1A). In three experiments, we noticed a small jump of CD69 around 8 to 9 days after the LM inoculation (Figs. 1A and S1A, showing the FACS plots in all five mice in this group. Fig. S1B shows the pool data of all three independent experiments). This phenomenon was inconspicuously shown in a report from another group without arousing any curiosity (Ciabattini et al., 2008). We decided to investigate whether this phenomenon could be recaptured *in vitro* and whether it had any relevance in regulation of T cells after their primary response. We stimulated OT-II cells *in vitro* with OVA, and the activated cells were harvested after 48 h by FACS purification (termed previously activated T cells, or PA T; the gradual downregulation of CD69 on these activated T cells upon FACS sorting is shown in Fig. S2A). These cells were then co-cultured in the absence of antigen with GM-CSF/IL-4-induced bone marrow DCs (BMDCs) or immortalized DC line DC1940 (Steiner et al., 2008). Intriguingly, a percentage of previously activated OT-II re-expressed CD69 and data are pooled from multiple experiments (Fig. 1B), although the response intensity was considerably lower than that to DC + OVA. Freshly isolated naïve OT-II CD4 T cells, however, did not show such an upregulation (Fig. 1C). This upregulation was absent in co-culture with B6 MEF or 3T3 cells (Fig. 1D). To test this phenomenon in the complete absence of antigen, we stimulated B6 CD4 T cells *in vitro* with anti-CD3ε and anti-CD28, and the resulting PA T cells were co-cultured with the stimulators used above. The CD69 upregulation was seen in these non-specifically activated CD4 T cells co-cultured with B6 splenic CD11c^+^ cells and DC1940 (Fig. 1E), and not with B6 MEF or 3T3 cells, and data are pooled from multiple experiments (Fig. 1F). It is also worth noting that T cells assayed here did not show significant cell death in this duration (Fig. S2B). Data in Fig. 1C–E are also pooled from multiple experiments and shown in Fig. 3A–C, respectively. These observations seem to suggest that PA T cells have a unique response to DCs following their primary activation and this response itself does not involve antigen specificity.

### PA T encounter with DCs results in limited activation indicated by upregulating of *Il2* and *Bcl2*

**Figure 2:**
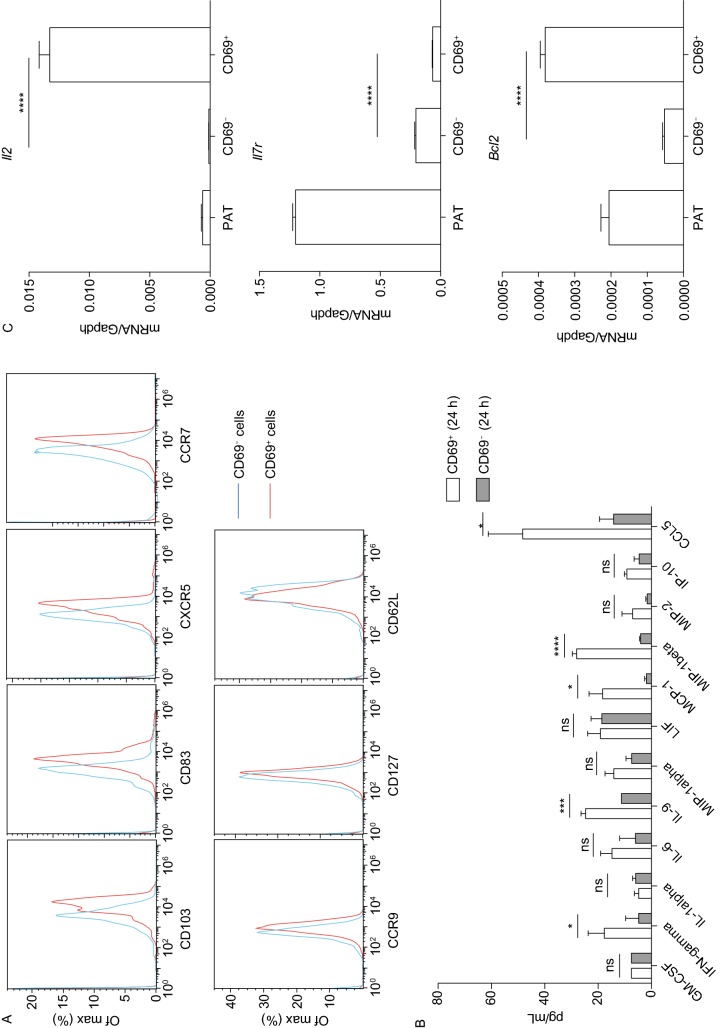
**PA T encounter with DCs results in limited activation involving upregulation of*Il2*and*Bcl2***. (A) PA T cells were co-cultured with DC1940 for 24 h, then CD4^+^ populations were gated into CD69^+^ and CD69^−^ cells by FACS. The expression of CD103, CXCR5, CD62L, CCR9, CCR7, CD127 and CD83 between CD69^+^ and CD69^−^ cells were compared. Three replicates in each group (*n* = 3), and results are representative of three independent experiments (*N* = 3). (B) CD69^+^ and CD69^−^ cells cultured in complete RPMI for 24 h without additional treatment, then the cytokines released to the supernatant were measured by ELISA. Pooled data from three independent experiments are shown. (C) Gene expression of *Il7r*, *Il2* and *Bcl2* was determined by QPCR in PA T cells or in CD69 positive or negative populations purified by FACS sorting. Three replicates in each group (*n* = 3), and results are representative of three independent experiments (*N* = 3). GAPDH was used for normalization

The stimulation provided by DCs to PA T cells led to some surface marker expression change, such as a slight up regulation of CD103, CXCR5 and CCR7 in CD69^+^ population, while CD127 (IL7R) appeared to be expressed at similar levels (Figs. 2A and S4). A screening analysis of cytokines in the culture supernatant indicated some differences, such as the increases in IFN-γ, CCL5 and several MIPs (Fig. 2B). In contrast to the nominal changes at the phonotypical level, *Il2* and *Bcl2* RNA in CD69^+^ T cells were increased, *Il7r* on the other hand appeared to be lower (Fig. 2C). Interestingly, *Il7r* mRNA level, low to begin with, was significantly reduced in CD69^+^ cells, yet this difference was not seen at the protein level (Fig. 2A). We do not know the reason behind this discrepancy. One possible explanation was the slightly better activated CD69^+^ cells allowed more efficient translation of this mRNA. This is certainly our unsubstantiated speculation and the issue was not analyzed further. Collectively, these results indicate that second encounter of antigen-free DCs invokes a very limited activation in these T cells that could be captured by IL-2 and CD69 analyses.

### PT-A reactivation is associated with IL-2 production by DCs

**Figure 3:**
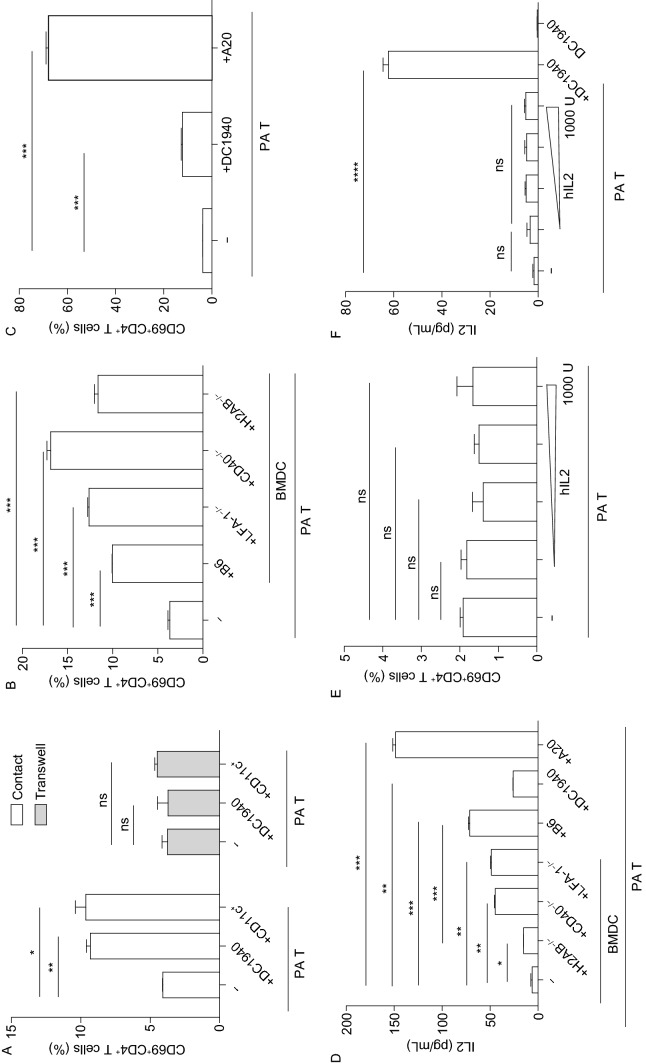
**Minimal PA T reactivation is associated with IL-2 production by DCs**. (A) PA T cells were co-cultured with DC1940 or CD11c^+^ splenocytes for 24 h in transwell assays, CD69 expression was analyzed and IL-2 secretion was detected. Three replicates in each group (*n* = 3), and results are representative of three independent experiments (*N* = 3). (B) Similar to (A), CD69 expression of PA T cells cocultured with BMDCs from WT, *CD11a*^−/−^*, Cd40*^−/−^ and MHC class II^−/−^ mice. Three replicates in each group (*n* = 3), and results are representative of three independent experiments (*N* = 3). (C) Similar to (A), CD69 expression on PA T co-cultured with DC1940 or A20 cell lines. Three replicates in each group (*n* = 3), and results are representative of three independent experiments (*N* = 3). (D) PA T cells were co-cultured with DC1940, A20, WT BMDCs and BMDCs from the indicated gene deficient mice. IL-2 in the supernatant was measured by ELISA. Three replicates in each group (*n* = 3), and results are representative of three independent experiments (*N* = 3). (E) CD69 expression and (F) IL-2 secretion as in (A) without DCs yet in the presence of increasing amounts of IL-2 (none, 100 U,200 U, 500 U and 1,000 U/mL) for 24 h. Three replicates in each group (*n* = 3), and results are representative of three independent experiments (*N* = 3)

To examine the essential requirements for this low-grade activation, we first tested the involvement of physical contact. Transwell assays showed that contact was critical for the partial CD69 expression (Fig. 3A). Yet, BMDCs from MHC class II, LFA-1 and CD40 deficient mice were fully capable of inducing the partial CD69 expression (Fig. 3B), dismissing the need of strong DC/T cell binding or CD40/CD40L crosstalk. In search for a potential non-surface protein-mediated effect, we noticed a striking phenotype that activated CD4 T cells expressed exceptionally high levels of CD69 following co-cultured with A20 B cell line (Fig. 3C). At the same time, all stimulatory co-cultures consistently produced low levels of IL-2, with the sole exception of the A20 culture where ample IL-2 was made (Fig. 3D), in line with A20’s strong ability to produce IL-2 reported in the literature (Kakiuchi et al., 1991). The higher response to A20 was not driven by a hidden allogenic response (A20 is of H2-d) as this upregulation of CD69 was also seen with bulk PA T cells activated by anti-CD3/CD28 which have a TCR pool vastly different from that of singular OT-II TCR (Fig. S5). In this experiment, we also found that MHC class II deficiency led to a decreased IL-2 production in contrast to the similar level of CD69 expression. It is possible that nominal IL-2 production by T cells in response to DC contact may require the basal TCR MHC binding; effects of this nature have been demonstrated in T cell survival and subtle growth advantage in homeostatic expansion (Kieper et al., 2004; Tanchot et al., 1997). On a whole, the result suggested that PA T reactivation may be linked to the IL-2 production by cells which they interact with. Interestingly, CD69 expression was not increased in the presence of high IL-2 alone (Fig. 3E). IL-2 production from the PA T was also not restored by exogenous IL-2 either (Fig. 3F), suggesting that IL-2 was delivered via surface contact.

### Surface delivery of IL-2 from DCs to PA T

**Figure 4:**
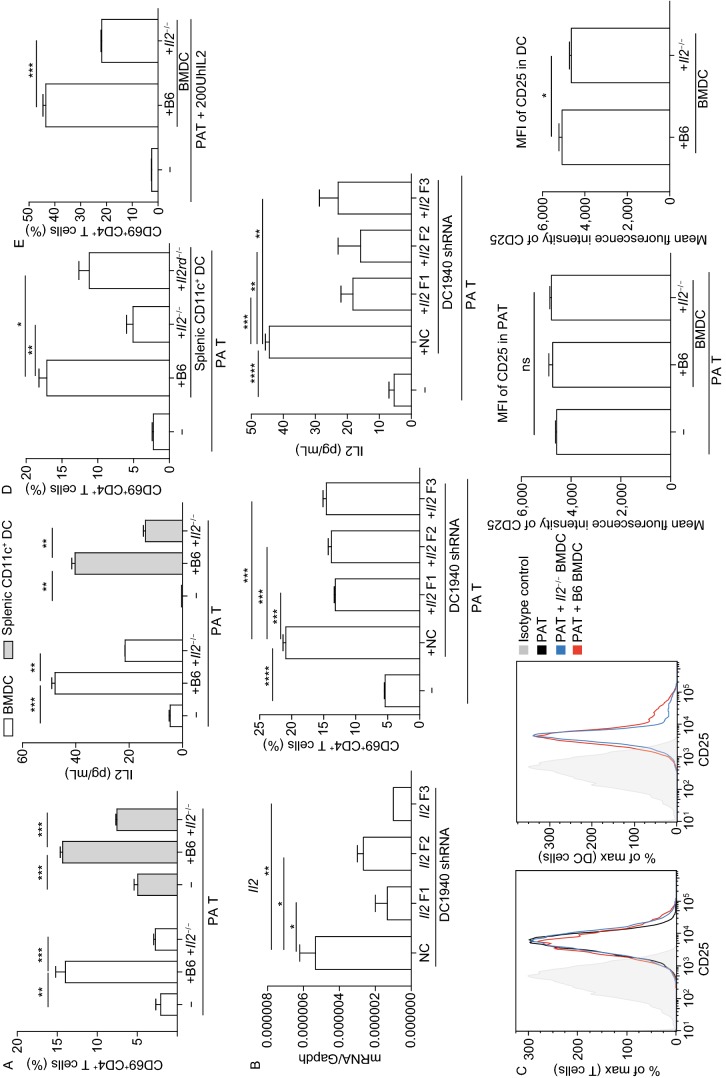
**Surface delivery of IL-2 from DCs to PA T**. (A) CD69 expression and IL-2 secretion of PA T cells co-cultured with BMDCs or splenic CD11c^+^ DCs from B6 and *Il2*^−/−^ mice for 24 h. Three replicates in each group (*n* = 3), and results are representative of five independent experiments (*N* = 5). (B) the efficiency of *Il2* knockdown by viral vector-delivered shRNA in DC1940 cells (upper). NC: control shRNA, F1, 2 and 3 are three independent shRNA sequences. CD69 expression (middle) and IL2 secretion (lower) by PA T co-cultured with IL-2 knockdown DC1940. Three replicates in each group (*n* = 3), and results are representative of three independent experiments (*N* = 3). (C) CD25 expression on PA T cells and BMDCs with or without co-culture. First column: T cells, and second: DCs. Three replicates in each group (*n* = 3), and results are representative of five independent experiments (*N* = 5). Next two columns: CD25 mean fluorescence intensity (MFI) is shown in PA T and DC groups. ns means no significant difference, *P < 0.05(Unpaired Student’s *t* test). (D) CD69 expression on PA T cells co-cultured with WT, *Il2*^−/−^ or *Il2rα*^−/−^ BMDCs. Three replicates in each group (*n* = 3), and results are representative of three independent experiments (*N* = 3). (E) CD69 expression on PA T cells co-cultured with WT or *Il2*^−/−^ BMDCs in the presence of added IL-2. Three replicates in each group (*n* = 3), and results are representative of five independent experiments (*N* = 5)

We used IL-2-deficient DCs to confirm the source of IL-2. Indeed, when these BMDCs were used in the co-culture assay, the CD69 expression and IL-2 production were reduced (Fig. 4A). Although our data do not rule out IL-2 production from T cells, it is clear that IL-2 from DCs was critical to start the “minimal” activation of the T cells, including the upregulation of CD69 in some cells and the potential contribution of IL-2 into the overall pool. To rule out any abnormality in the IL-2-deficient DCs, shRNA was used to knock down IL-2 in DC1940 (Fig. 4B). Again, the CD69 expression and IL-2 present in the culture supernatant were both reduced. PA T expression of CD25 changed little with or without co-culture with DCs and data are pooled from multiple experiments (Fig. 4C). We therefore considered if CD25 on the DC surface was able to transport IL-2 to CD4 T cells. Indeed, in this co-culture system, CD25 expression on WT DCs was higher than KO (Fig. 4C). This expression may be contributing to the delivery of IL-2 to PA T. We cultured PA T cells with CD25-deficient/IL-2-sufficient BMDCs. The result shows that in this co-culture, similar to IL-2-deficient DCs, CD69 expression on CD4 T cells was reduced (Fig. 4D), suggesting at least a sizable part of IL-2 was delivered from DC to PA T via cell surface CD25. In this co-culture system, CD69 expression was not completely restored by exogenous IL-2 in IL-2-deficient BMDCs (Fig. 4E), stressing the need for a cognate origin of both IL-2 and CD25. It should be noted that a fraction of A20 cells, by our own analysis, also expresses CD25. To provide a direct comparison, we performed an experiment comparing these two types of DCs in the presence or absence of added IL-2 (Fig. S6). The results show that while CD69 expression was higher with extrinsic IL-2, the relative difference between WT and IL-2^−/−^ DCs remained.

### IL-2^+^DC contact leads to an altered gene expression profile

**Figure 5:**
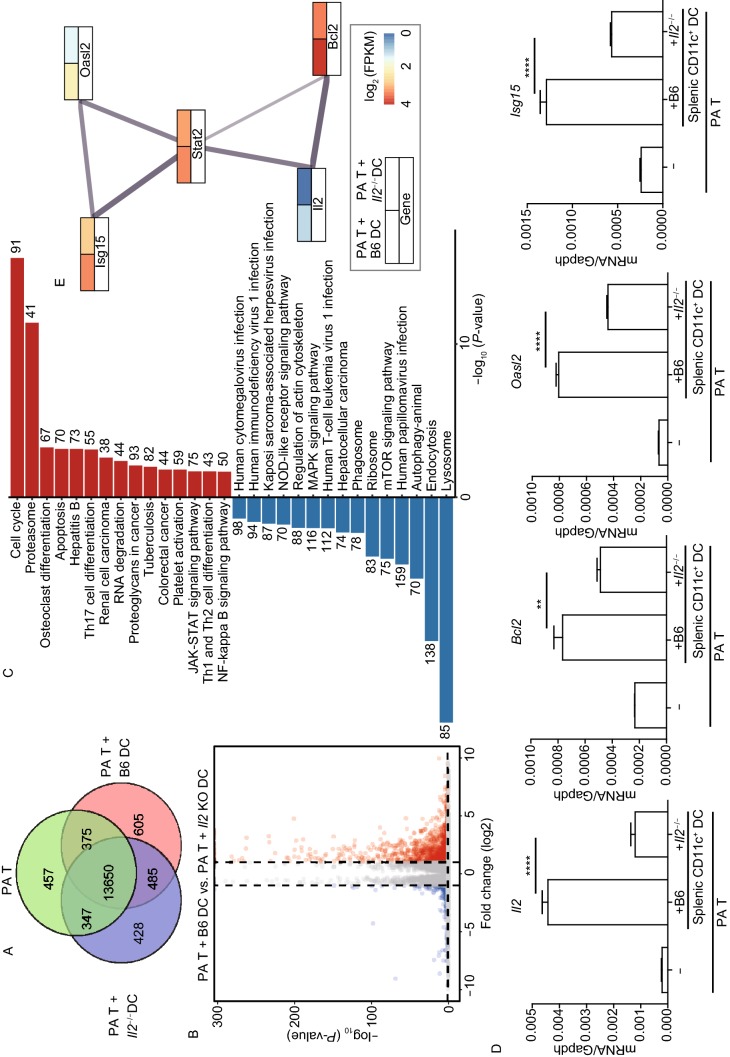
**IL-2**^**+**^**DC contact leads to an altered expression profile**. Differential gene expression by PA T cells co-cultured with B6 or *Il2*^−/−^ splenic CD11C^+^ in PA T cells. These PA T cells were purified by FACS, and mRNA was prepared for RNA-sequence analysis as described. (A) Venn diagram of number of genes differentially expressed in PA T, PA T + *Il2*^−/−^ DC and PA T + B6 DC. A total of 13,650 genes were detected in the three conditional PA T cells. The non-overlapping part is the number of genes present in only one experimental group. (B) Volcano plot depicts differentially expressed genes (DEGs) between PA T + B6 and PA T + *Il2*^−/−^ DCs. Red dots are genes that are significantly up regulated genes in PA T + B6 DCs, while blue dots are significantly down regulated. The horizontal dashed line means *P* = 0.05. (C) Enriched KEGG pathways for differentially expressed genes between PA T + B6 and PA T + *Il2*^−/−^ DCs. Terms that are enriched from upregulated genes in PA T + B6 DC are marked by red, and terms enriched from down regulated genes are marked blue. The number of associated genes in each GO categories is presented around the bar (*P*-value <0.05). (D) mRNA level of *Il2*, *Bcl2*, *Oasl2* and *Isg15* in PA T, PA T + B6 and PA T + *Il2*^−/−^ DCs. The reference is GAPDH. Results are representative of at least of 3 independent experiments. *N* ≥ 3 for all analyses. (E) Heatmap for *Isg15*, *Oasl2*, *Stat2*, *Il2* and *Bcl2* differentially expressed between PA T + *Il2*^−/−^ DC and PA T + B6 DCs. Lines represent protein-protein associations, and the thicker the edge is, the stronger the association should be. Bottom: the left frame is the expression level from PA T + B6 DCs, while the right stands for that from PA T + *Il2*^−/−^ DC. Red color indicates a higher expression, while the blue color indicates a lower expression

We performed RNA-seq analysis and found that PA T, and PA T cells co-cultured with B6 splenic CD11C^+^ DCs (PA T + B6 DC) and IL-2-deficient DCs (PA T + *Il2*^−/−^ DC) had substantial differences in gene expression profile (Fig. 5A). We next focused on the genes that were expressed differentially in PA T + B6 DC and PA T + *Il2*^−/−^ DC by Volcano plot (Fig. 5B). In pair-wise comparison between B6 and IL-2-deficient DC co-cultured PA T cells, genes expression and their functional pathway are shown (Fig. 5C). The result showed the statistically most significant differences in cell cycle and apoptosis. Previous reports found that (Zhou et al., 2017; Park et al., 2000; Smith, 2015; Yang et al., 1997) *Il2*, *Bcl2*, *Stat2*, *Isg15*, *Oasl2* may participate in the cell survival and anti-apoptosis processes, and these genes were found to be quantitatively different in the culture with B6 DCs and IL-2-deficient DCs by real time PCR (Fig. 5D). Gene association analysis showed that these associated with type 1 interferon responses were heavily interconnected (Fig. 5E).

### IL-2-positive DC coculture promotes activated CD4 T cells into a long lasting population *in vivo*

**Figure 6:**
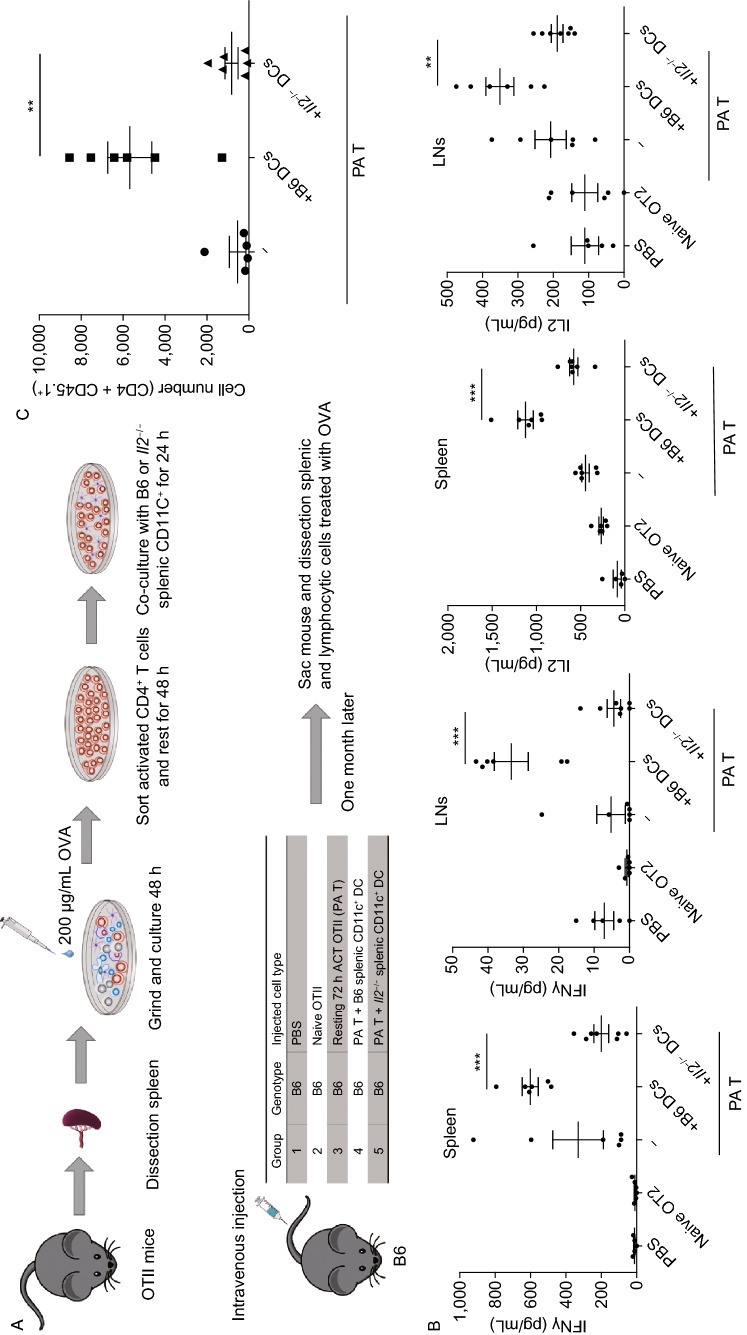
**IL-2-positive DC coculture promotes PA T cells’ survival*in vivo***. (A) PA T cells were prepared *in vitro*, shown as the flow chart, then the cells were i.v. injected into 6–8 week old C57BL/6 wild type mice, 1 × 10^6^ cells per mouse (5 per group). Experimental group mice were injected with resting PA T cells, PA T co-cultured with WT or *Il2*^−/−^ splenic CD11c^+^ DCs. Mice injected with PBS were the negative control. (B) After one month, the cells from spleen and LNs were collected, and in 24-well-plate 1 × 10^6^ or 2 × 10^6^ cells added to 2 × 10^6^ feeder population of irradiated B6 splenocytes which were prior-treated with 3,000 rad γ irradiation. The clonal expansion experiment was carried out *in vitro* by adding soluble OVA. IFN-γ and IL-2 in the supernatant were measured by ELISA. Every dot stands for one mouse. Results are representative of two independent experiments. (C) PA T cells from CD45.1^+^ OT-II mice were prepared *in vitro*, then the cells were i.v. injected into 6-8 weeks CD45.2^+^C57BL/6 wild type mice, 1 × 10^6^ cells per mouse. Experimental group mice were injected with resting PA T cells, PA T co-cultured with B6 or *Il2*^−/−^ splenic CD11c^+^ DCs. After three months, the host mice were re-challenged by injected 50 µg OVA and 10 ug LPS per mice, 72 h later, the cells from spleen and LNs were collected, then analyzed for the total cell number of CD4^+^CD45.1^+^ cells in host mice by FCAS. Every symbol stands one mouse. Results are representative of two independent experiments. **P < 0.01, ***P < 0.001 (Unpaired Student’s *t* test)

**Figure 7:**
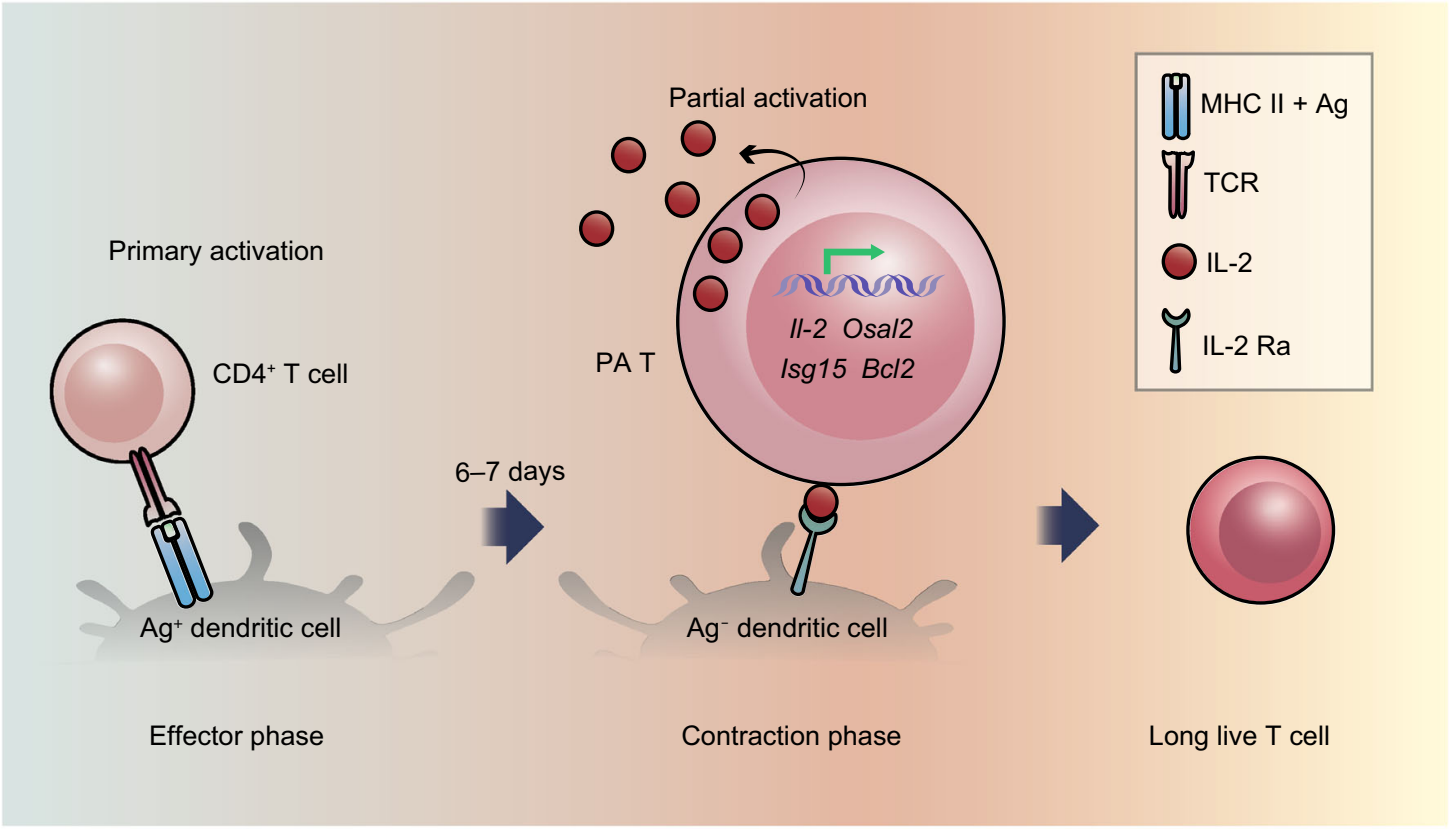
**The proposed programing in PA T after DC contact**. Schematic overview of the interplay between IL-2, DCs, previously activated CD4 T cells and memory-like CD4 T cells. At the beginning of contraction phase, previously activated CD4 T cells in a temporally limited window can interact with antigen-free DCs. These DCs produce small amounts of IL-2 that is presented on their surface via DC-intrinsic CD25. IL-2 so delivered initiates a mild activation programing in PA T cells to produce small amounts of IL-2 and CD69 expression in a sub population of the latter. This programing, in addition to support IL-2 production, also enhances the expression several Type I IFN-related and anti-apoptotic genes, for long term survival of these CD4 T cells

To determine whether the low-grade activation of PA T cells had any impact on their secondary responses *in vivo*, we transferred these T cells into naïve recipients, and spleens and LNs were harvested after one month for restimulation with DC pulsed with soluble OVA (Fig. 6A scheme). Compared with untreated or IL-2-deficient DC-treated PA T cells, those treated with WT DCs showed stronger IFNγ and IL-2 production, suggesting a stronger secondary response (Fig. 6B). To directly compare the cell number of infused T cells, we transfused CD45.1 PA T with or without DC treatment into the recipient mice. Three months later, those mice were challenged with OVA plus LPS, and the CD45.1-positive T cells were counted three days later. Again, the prior encounter with IL-2-sufficient DCs significantly boosted the number counts in comparison with the *Il2*^−/−^ DCs (Fig. 6C). These results indicate that the minimalist activation, while resulting in no overt phenotypic changes in these CD4 T cells, altered the gene expression and was conducive to a prolonged T cell survival in the host (Fig. 7, proposed programing in PA T).

## Discussion

IL-2 production by DCs was first revealed by Granucci et al., with the advent of gene expression microarray (Granucci et al., 2001), and it was reported at that time to play a role in both CD4 and CD8 T cell proliferative responses to allogenic DCs. It was later reported by the same group that microbial products, including common PAMPs such as GpG, peptidoglycan and LPS, drove the IL-2 production. The production could be tuned by the CD40 signaling originated from activated T cells as well (Granucci et al., 2003). At least for Dectin-1-mediated activation, IL-2 transcription was controlled via NFAT in phagocytes (Goodridge et al., 2007). However, this notion of DC-originated IL-2 being critical for primary T cell activation was questioned by another report (Schartz et al., 2005). Of particular interest, in the latter report, adoptive transfer of antige-loaded IL-2-sufficient and deficient DCs did not result in any difference in CD8 memory response. The setup was meant to address IL-2 production by DCs at the primary activation of T cells. An inconspicuous oversight of that report, in light of our work here, was whether the IL-2 expression in recipient resident DCs was involved as an environmental and temporally discreet factor to support T cell memory beyond initial DC/T cell interaction (Schartz et al., 2005). Therefore, it remains premature to dismiss the impact of DC-intrinsic IL-2 production in the totality of T cell biology.

Regarding the role of IL-2 in T cell memory development, one unsettled issue is the timing of bioavailability. IL-2 production has been found to be rapid and transient (Sojka et al., 2004). This pattern is well explained from the negative feedback in the environmental milieu (Villarino et al., 2007). In human, IL-2 production peaks in day one and becomes undetectable on day three or four, to be followed by a more sustained T cell proliferation (Venuta et al., 1983). This certainly raises the issue of timing as the IL-2 suggested to be mandatory in late phase of primary activation is not available from the same T cells unless intervened otherwise. Our results seem to draw such a scenario: T cells expand in the presence of antigen with a robust IL-2 production to support the proliferation. The IL-2 production is rapidly reduced thereafter in preparation of the contraction from the peak T cell activation. At this moment, a percentage of DCs by coincidence bind to DCs in the absence of antigen. The IL-2 produced in these DCs triggers a secondary IL-2 production in these previously activated CD4 T cells to mediate a program of cell survival. Due to the transient nature of DC/T cell contact, another possibility is that T cells that have accumulated sufficient number of contacts with DCs venture to enter the second round of IL-2 production. The probabilistic nature of the DC/T cell contact may also limit a large number of T cells from entering memory programing, providing a crude selection filter at this moment. Regarding the observation that DC CD25 appears to contribute to the subtle T cell activation, one possible involvement is its role in bringing IL-2 to the DC/T cell contact interface, as T cells at this stage do not express this high affinity receptor. We have shown in this report that extrinsic addition of IL-2 was not as effective. In reality, it would be extraordinarily difficult to sustain IL-2 at high levels days after the primary activation. Low amounts of IL-2 would in theory require some degree of assistance for its efficient delivery to T cells. Previous work on several cytokines seem to point to a shared mechanism. In TH-17 programing, IL-6 is presented on DCs via IL-6Rα chain for highly efficient activation of T cells (Heink et al., 2017). In that report, one theory is the formation of some type of signaling complex at the contact site across two cells. However, the topography of combining cytokine and multiple receptor signaling chains in such a configuration has not been formally proposed. In Daclizumab treatment, a humanized monoclonal antibody used to treat inflammation, IL-2 was also presented by CD25 on DCs for robust signaling in T cells (Wuest et al., 2011). Our work again suggests the utilization of this special method of cytokine delivery. Why this way of cytokine delivery is superior to external loading is not clear. One possibility is the pre-existing CD25/IL-2 complex formation inside DCs, leading to a more sustained/stable presence on the DC surface. On the other side of DC/T cell pair, whether the subtle PA T reactivation induced gene profile changes are a consequence of this particular mode of cytokine/receptor arrangement or determined by other temporal factors or ligand density would require more sophisticated setups. IL-2 activates STAT5A/B and to some degree also signals via STAT1 and STAT3. The JAK-STAT pathway is critical for the differentiation of T cells subsets (Spolski et al., 2017). Whether a hard to detect, miniature signal in the contraction state of T cell activation also regulates the latter long term survival is a worthy follow-up study. Lastly, we cannot rule out epigenetic changes in DCs that are deficient in IL-2/CD25. Although these proteins are not known to directly regulate DC development or phenotype, strong evidence suggests that IL2/CD25 (via STATs and IRFs)-associated signaling is a part of DC programming (Tian et al., 2017). These differences themselves may also explain why the lack of these two factors leads to an altered T cell survival upon DC/T encounter.

We must stress that results in this work are mostly suggestive pertaining to T cell memory. First, we used an *in vitro* model to simulate a potential *in vivo* process. Whether the CD69 upregulation seen in the late phase of T cell activation is related to our observation is unknown. Second, verifications will be needed to show that the *in vivo* CD69^+^ population has the survival advantage in real life T cell response. We therefore do not advocate a direct link between our findings and *in vivo* DC/T cell behavior, let alone the precise role of IL-2 from DCs in T cell memory formation. This report may be of some value in providing the missing link regarding the IL-2 availability during the late phase of primary CD4 activation that is conducive to memory formation. While this finding is intriguing, advanced animal work will provide more precise insight. For instance, a timed or activation stage-triggered IL-2 deletion in DCs, or a detailed analysis of accumulation DC/PA T binding will be highly informative. In addition to IL-2 production in the T cells, this event appears to regulate several groups of gene expression. Whether and how much these gene regulations individually and collectively contribute to IL-2 production or even to the memory development should present intriguing topics for future work.

## Materials and methods

### Mice

All mice were in C57BL/6 background. WT, OT-II, CD11a^−/−^, IL-2RA^−/−^, CD40^−/−^ CD45.1 congenic mice were bred and housed at Tsinghua University Animal Facilities. IL-2RA^−/−^ were made in house with CRISPR/Cas9 techniques and verified by genotyping. IL-2^−/−^ mice were purchased from JAX. H2-IAb (H2AB) deficient mice (MHC class II^−/−^ mice) were a gift from Dr. Hai Qi of Tsinghua University. The Animal Experiments Committee of Tsinghua University approved all of the experiments reported in this study.

### Production of Cas9 mRNA and sgRNA

CRISPR/Cas9 vectors were gifts from Dr. Hai Qi of Tsinghua University. Vector pST1374-N-NLS-flag-linker-Cas9 expressing cas9 was digested with *Age*I (NEB) and *Spe*I (NEB), and the linearized vector was gel purified, and used as the template for *in vitro* transcription using mMESSAGE mMACHINE T7 ULTRA kit (Life Technologies, AM1345). Vector pUC57kan-T7-gRNA expressing sgRNA was digested with *Dra*I (NEB), and the linearized vector was gel purified. A pair of oligos for targeting *Il2ra* was annealed, phosphorylated, and ligated to the linearized vector, and used as the template for *in vitro* transcription using MEGAshortscript T7 Transcription kit (Life Technologies, AM1354). Both the Cas9 mRNA and the sgRNA were purified using MEGAclear kit (Life Technologies, AM1908) and eluted in RNase-free water, and stored in −80 °C. sgRNA-Il2ra: 5′-TAGGGAACCATAGTACCCAGTTGTC-3′; and 5′-AAACGACAACTGGGTACTATGGTTC-3′

### Intracytoplasmic RNA microinjection

The intracytoplasmic RNA microinjection was performed at Tsinghua University Animal Facilities. Each embryo was microinjected with 25 ng/µL sgRNA and 50 ng/µL Cas9 mRNA into the cytoplasm. After microinjection, surviving embryos were implanted on the same day into oviduct of pseudo pregnant C57BL/6 female mice. Full-term pups were obtained by natural labor at 19.5-day post coitum (dpc).

### Genotyping of mice

All mice were genotyped with the tail DNA. Two pairs of primers were used to identify the IL-2RA^−/−^ mice. Primer Il2ra-WT (5′-CATTAACCATAGTACCCAGTT-3′) and common-R (5′-GCAAAGCCAAACCATCCCTG-3′) can detected the wild type anneal. The product size was 305 bp. Primer Il2ra-M (5′-CATTAACCATAGTACCCAGTG-3′) and common-R can detected the mutant anneal. The product of PCR was 304bp. The PCR reaction conditions were as follows: 95 °C, 5 min; 95 °C, 30 s, 58.5 °C, 30 s, 72 °C, 30 s, 30 cycles; 72 °C, 10 min.

### Antibodies and reagents

Antibodies for FACS, Vα2 TCR chain, CD69, CD4, CD25, CD127, CXCR5, CCR7, CCR9, CD62L, CD83, CD45.1 and CD103, and ELISA kits were purchased from eBioscience. Others are as follows, anti-mouse CD3e (eBioscience, 85-16-0032-85), anti-mouse CD28 (eBioscience, 85-16-0281-85), murine GM-CSF (Peprotech, 315-03-1000), murine IL-4 (Peprotech, 214-14-1000), human IL-2 (Peprotech, 200-02-1000), anti-mouse CD4 APC (eBioscience,17-0041-82), rat IgG2b K isotype control PE (eBioscience, 12-4031-82), rat IgG2b k isotype control APC (eBioscience, 17-4031-82), rat IgG2b k isotype control FITC (eBioscience, 11-4031-82), anti-mouse V alpha 2 TCR eFluor 450 (eBioscience, 48-5812-82), anti-mouse CD69 APC (eBioscience, 17-0691-82), anti-mouse V alpha 2 TCR PE (eBioscience, 12-5812-82), anti-mouse CD103 (Integrin alpha E) APC (eBioscience, 17-1031-82), anti-mouse CD197 (CCR7) PE (eBioscience, 85-12-1971-80), anti-mouse CD199 (CCR9) PE (eBioscience, 85-12-1991-80), anti-mouse CD83 APC (eBioscience, 17-0839-41), anti-mouse CD45.1 PE (eBioscience, 12-0453-82), anti-mouse CD62L FITC (eBioscience, 85-11-0621-82), PerCP/Cy5.5 anti-mouse CD127 (BioLegend, 135021), PE anti-mouse CD25 (BioLegend, 102007), PE anti-mouse CD185 (CXCR5) (BioLegend, 145503), mouse IL-2 ELISA Set (eBioscience, 555148), mouse IFN γ ELISA Ready-SET-Go (eBioscience, 85-88-7314-77), 1× TMB solution (eBioscience, 00-4201-56), cell lines and primary cell culture DC1940 (a gift from Dr. Li Wu of Tsinghua University), A20, 3T3 and MEF were cultured in complete RPMI1640 (GIBCO) supplemented with 10% FBS (GIBCO), 100 U/mL penicillin, and 100 mg/mL streptomycin,10 mmol/L HEPES and 50 µmol/L β-mercaptoethanol. Bone marrows were differentiated for 6 days with 20 ng/mL GM-CSF and 10 ng/mL IL-4 in complete RPMI medium to produce BMDCs respectively. On day 6, BMDCs which were grown in 24-well plate were used directly for experiments. 293FT cells were cultured in DMEM (GIBCO) supplemented with 10% FBS. All cells were cultured at 37 with 5% CO_2_.

### Isolation of primary cells

Primary CD4 T cells were isolated from OT-II or B6 mice splenocytes by EasySep Mouse CD4^+^ T cell Isolation Kits (Stem Cell, 19852) and sometimes sorted by FACS with an anti-Vα2 TCR antibody. Primary CD11c DC cells were isolated from B6 or different gene knockout mice splenocytes by EasySep Mouse CD11c positive selection kit II (Stem Cell, 18780). Primary cells were obtained according to the protocol of the kits.

### Co-culture assay

In our study, we treated OT-II mice splenocytes with 200 µg/mL OVA about 48 h, and isolated activated CD4^+^CD69^+^ T cells by FACS with anti-mouse Vα2 TCR and anti-mouse CD69 antibodies. Then the PA T cells were harvested. Sometimes we treated B6 splenocytes with anti-mouse CD3e and anti-mouse CD28 for about 48 h. PA T cells were cultured in complete RPMI1640 supplemented with 10% FBS, 200 U/mL penicillin, and 200 mg/mL streptomycin, 10 mmol/L HEPES and 50 µmol/L β-mercaptoethanol. After resting for 48 h, PA T cells were co-cultured with different kinds of DCs or cell-lines. In 24-well-plate co-culture assay *in vitro*, the ratio of PA T and DCs is 2 to 1 (2 × 10^5^ and 1 × 10^5^ cells). Co-culture was carried out for 24 h, PA T cells or DCs were harvested to detect surface markers. The supernatants were stored in −20 °C for testing IL-2 or IFN-γ secretion by ELISA.

### The transwell assay

HTS Transwell-24-well permeable support with 0.4 µm Pore Polycarbonate Membrane and 6.5 mm Inserts, Sterile (Corning, 3396) were used in the transwell assay. In the transwell co-culture system, PA T cells were seeded in the upper layer, and DCs in the substrate. PA T cells were cultured at a 2:1 ratio with DCs for 24 h.

### Gene knockdown in DC1940 cell-lines

Lentiviral plasmids encoding targeting *Il2* shRNA or non-targeting shRNA (SHC002) were purchased from Sigma and the *Il2* shRNA TRC numbers were as follows: TRCN0000067198, TRCN0000067200, TRCN0000067202. The lentivirus was produced by co-transfection of 293FT cells with lentivirus expression vector, pCMV-VSV-G and pCMV-dR8.91 using Lipofectamine 2000 (Invitrogen, 11668027). DC1940 cells were infected with lentivirus at 500 ×*g*, 32 °C for 1.5 h. DC1940 cells were selected with puromycin (1 µg/mL) 72 h post-infection. The knockdown efficiency was analyzed by real-time PCR.

### Real-time PCR

RNA samples were prepared using TRIzol reagent (Invitrogen, 15596018) and first strand cDNA was synthesized with RrimeScript^TM^ RT-PCR Kit (TAKARA, RR047A). Real-time PCR was performed using 2 X RealStar Power SYBR mixture (GenStar, A311-10) in a BIO-RAD CFX96 Real-time System. *GAPDH* was used for normalization. The primer sequences were as follows: *Il2*, 5′-TGAGCAGGATGGAGAATTACAGG-3′ and 5′-ATGTGTTGTCAGAGCCCTTTAG-3′; *Oasl2*, 5′-AAACAGCTGAAGGGAGACCG-3′ and 5′-CTCGCTGCTGTACATTCCA-3′; *Isg15*, 5′-AGCAATGGCCTGGGACCTAAAG-3′ and 5′-TAAGACCGTCCTGGAGCACTG-3′; *IL-7 receptor* (*IL-7r*), 5′-ACAAGAACAACAATCCCACAGAG-3′ and 5′-TCGCTCCAGAAGCCTTTGAAG-3′; *Bcl2*, 5′-TGTGTGGAGAGCGTCAACAG-3′ and 5′-CAGACATGCACCTACCCAGC-3′; *GAPDH*, 5′- ATCAAGAAGGTGGTGAAGCA-3′ and 5′-AGACAACCTGGTCCTCAGTGT-3′.

### Sampling and RNA extraction for RNA-seq

We treated OT-II mice splenocytes with 200 µg/mL OVA about 48 h, and isolated activated CD4^+^CD69^+^ T cells by FACS with anti-mouse Vα2 TCR and anti-mouse CD69 antibodies. Then PA T cells were harvested, and cultured in culture media. After resting 48 h, PA T cells were co-cultured with B6 or IL-2^−/−^ CD11c^+^ DCs, which had been isolated from B6 or IL-2^−/−^ splenocytes by EasySep Mouse CD11c Positive Selection Kit II. PA T cells were cultured in complete RPMI1640 alone as a control. After 24 h, these T cells were harvested by FACS with Vα2 TCR^+^CD11c^−^. The total RNA of each sample was isolated using TRIzol reagent (Invitrogen). RNA quality and concentration were determined using NanoDrop 2000 (Thermo Scientific). The RNA samples were stored in −80 °C, and later sent to BGI (China) for RNA sequencing.

### Analysis of RNA sequencing

Sequencing was performed on a BGISEQ-500 RS unit using single-end sequencing, and averagely 23,656,293 raw sequencing reads were generated. Then we removed reads 1) with adaptors; 2) in which unknown bases were more than 10%; 3) having more than 50% bases in low quality (quality is no more than 5). A total of 23,593,592 clean reads were obtained. The fastq files were aligned to hg19 with HISAT2, with the following parameters: –phred64 -sensitive -I1 -X 1000.

### Transcriptome analysis

After alignment, the read counts for each gene were calculated by RSEM, and the expression values of each gene were normalized using FPKM.

DEGs were screened using Poisson distribution as in Audic and Claverie (1997). Denoting the number of unambiguous clean tags from gene A as x, given every gene’s expression occupies only a small part of the library, x yields to the Poisson distribution:

$$p\left( x \right) = \frac{{e^{ - \lambda } \lambda^{x} }}{x!}$$

The total clean tag number of the sample 1 is $N_{1}$, and the total clean tag number of sample 2 is $N_{2}$; gene A holds x tags in sample 1 and y tags in sample 2. The probability of gene A expressed equally between two samples can be calculated with:

$$p\left( {y|x} \right) = \left(\frac{N_{2}}{N_{1}}\right)^{y} \frac{{\left( {x + y} \right)!}}{{x!y!\left( {1 + \frac{{N_{2} }}{{N_{1} }}} \right)^{{\left( {x_{1} y_{1} } \right)}} }}$$

DEGs were then visualized by volcano plots, in which fold changes are measured by

$$log_{2} \frac{{FPKM_{1} }}{{FPKM_{2} }}$$

### DEG analysis

KEGG was used to perform pathway enrichment analysis of DEGs, which was automated by clusterProfiler. The calculated *P*-values were then corrected by Bonferroni’s method and a threshold of 0.05 was set. Heatmap (log2 scaled) was plotted with pheatmap, and protein-protein associations were analyzed by STRING (von Mering et al., 2003).

### Mouse multiplex cytokine assay

PA T cells were co-cultured at a 2:1 ratio with DC1940 cells 24 h, then CD69^+^ and CD69^−^ cells were obtained by FACS with anti-mouse Vα2 TCR and anti-mouse CD69 antibodies. These sorted cells were cultured 1 × 10^5^ per well in 24-well-plate in complete RPMI1640 respectively. The supernatants were collected at different time points (15 h and 24 h) and stored in −20 °C for mouse multiplex cytokine detection. According to the manufacture’s protocol (Millipore, MCYTOMAG-70K, MCYTOMAG-70K-PMX, MCYTMAG-70K-PX32), the secretion of cytokines was detected by Luminex.

### *Listeria monocytogens* infection

*Listeria monocyte* expressing ovalbumin (LM-OVA) were a gift from Dr. Chen Dong of Tsinghua University. Frozen stocks of LM-OVA were thawed and then diluted in fresh brain heart infusion (BHI) medium (BD) to reach mid-log growth phase. 8-week-old mice were intravenously injected via the lateral tail vein with sub-lethal (5 × 10^4^ CFUs) dose of LM-OVA that was suspended in 200 µL PBS per mouse. After 4 h, spleens and lymph nodes were collected as day 0 samples. At day 1–10 days after infection, spleens and LNs were collected and dissociated in PBS containing 0.05% Triton X-100, and bacterial CFUs were determined by plating on BHI agar plates. The CD4^+^ T cells in spleens and LNs were analyzed for CD69 expression by FACS.

### Adoptive transfers and re-stimulation clonal expansion experiments

After resting 48 h, PA T cells were co-cultured at a 2:1 ratio with B6 or IL-2^−/−^ CD11c^+^ DCs. PA T cells were cultured in complete RPMI1640 alone as a control. After 24 h, these T cells were harvested by FACS with Vα2 TCR^+^CD11c^−^. These PA T cells were transferred 1 × 10^6^ per mice into naïve C57BL/6 mice via intravenously. After one month, spleens and LNs were collected, 1 × 10^6^ or 2 × 10^6^ cells were seeded with 2 × 10^6^ feeder cells and treated with 100 µg/mL OVA for 48 h to 96 h in 24-well-plate. The feeder cells were pre-treated X-Ray with 3,000 rad radiations (RS 2000 Pro). The culture supernatants were collected and stored −80 °C for IFN-γ and IL-2 secretion analysis by ELISA.

### Statistics

Number of experimental repeats are shown in the figure legend. All bar graphs are means with SEM. Statistical analysis was performed with Student’s *t* test in GraphPad Prism software. *P* value < 0.05 was considered significant. *P < 0.05, **P < 0.01, ***P < 0.001, ****P < 0.0001. ns, not significant.

## Electronic supplementary material

Below is the link to the electronic supplementary material.

Supplementary material 1 (PDF 5956 kb)
