# Supplementary material for: Contact-dependent delivery of IL-2 by dendritic cells to CD4 T cells in the contraction phase promotes their long-term survival
Source: Protein Cell. 2019 Nov 5;11(2):108–23. doi: 10.1007/s13238-019-00662-0 (PMC6954898; doi:10.1007/s13238-019-00662-0)
Supplement: Supplementary file 1 — Supplementary material 1 (PDF 5956 kb) [file 13238_2019_662_MOESM1_ESM.pdf]

Supplemental Figure 1

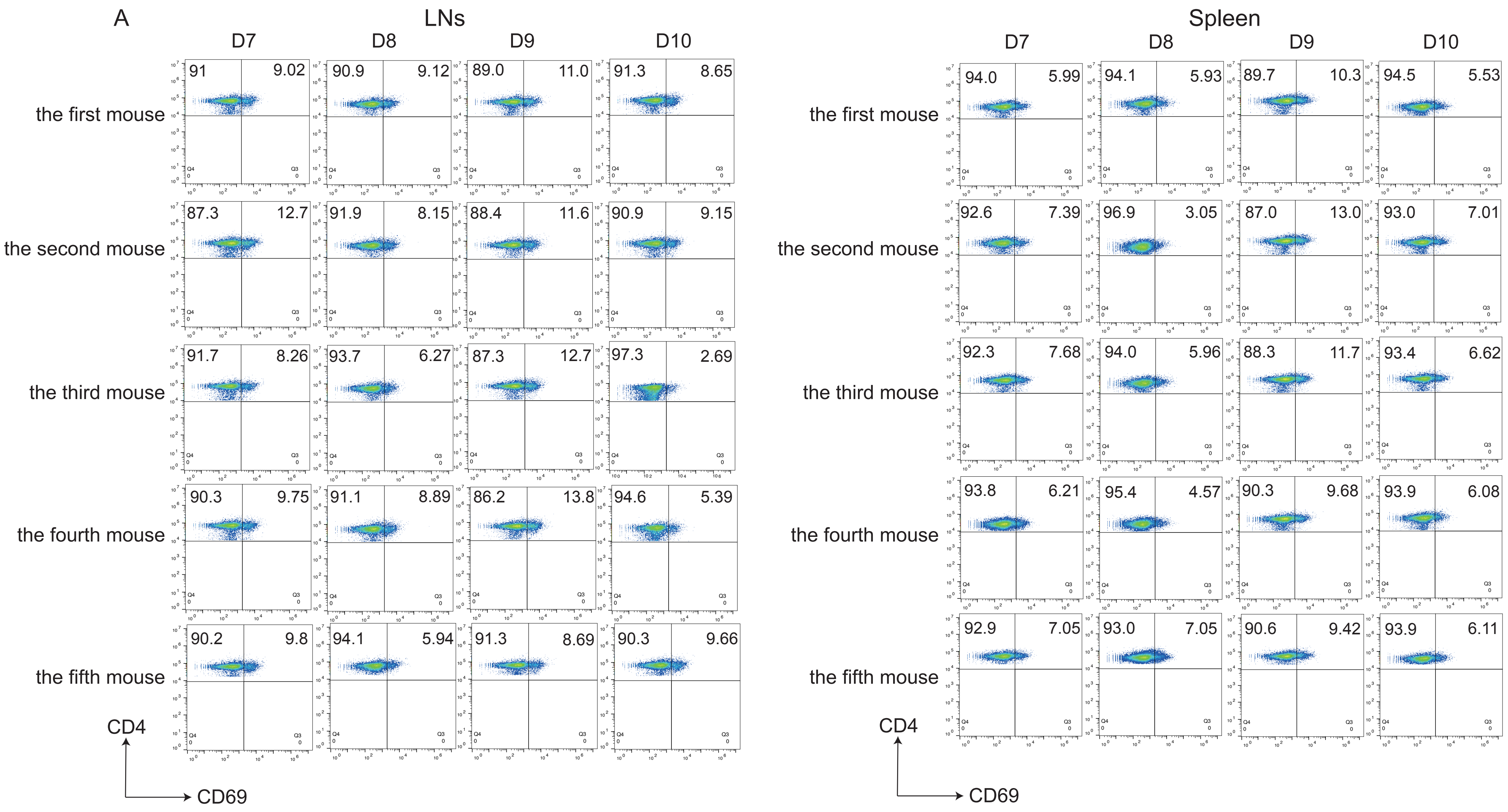

Supplemental Figure 1

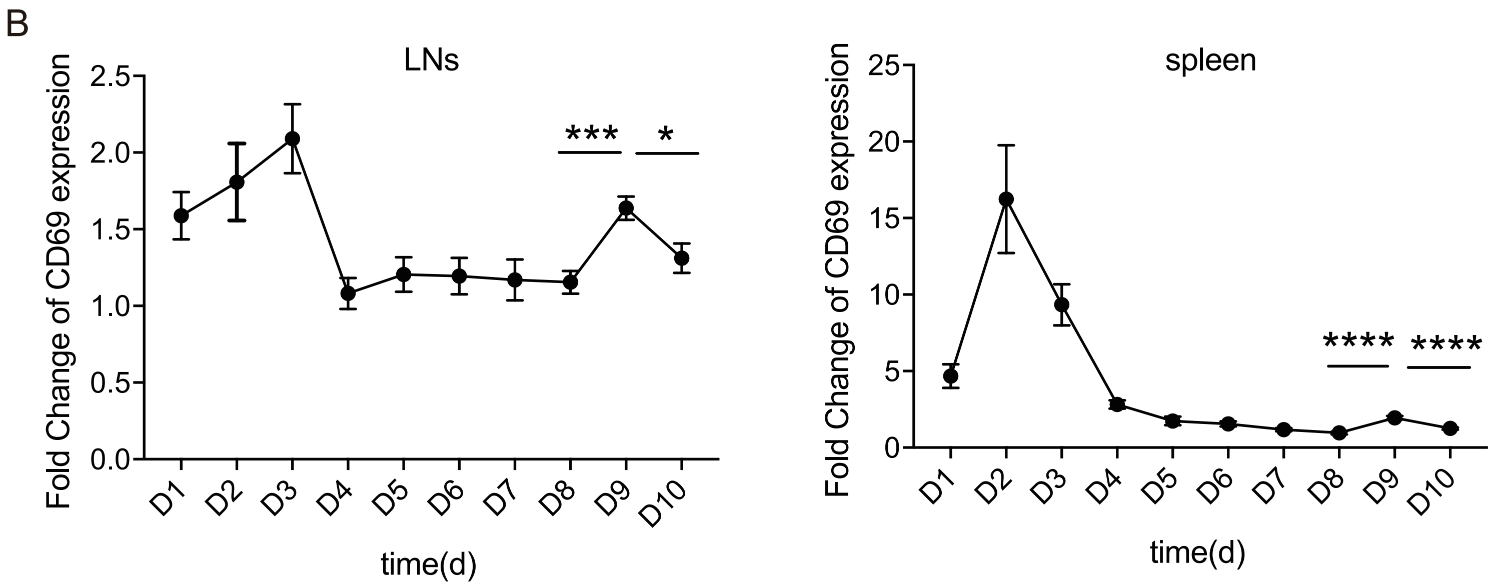

Figure 1. PA T cells upregulate CD69 in vivo
OT-II mice were i.v. injected with 0.1LD50 LM-OVA. dLNs (draining LNs) and spleen were harvested on stated days and CD69 expression on CD4 T cells as a percentage was determined by FACS. (A) Change of CD69 expression on T cells from five mice over day 7 to 10 in one independent experiment. (B) Pooled all data from three independent experiments are shown. Normalized CD69 expression of dLNs and spleen by D0 in three independent experiments (11 mice each data point), analyzed daily for fold change in CD69 expression. *$p<0.05$, ***$p<0.001$, ****$p<0.0001$ (Unpaired Student's t test).

Supplemental Figure 2

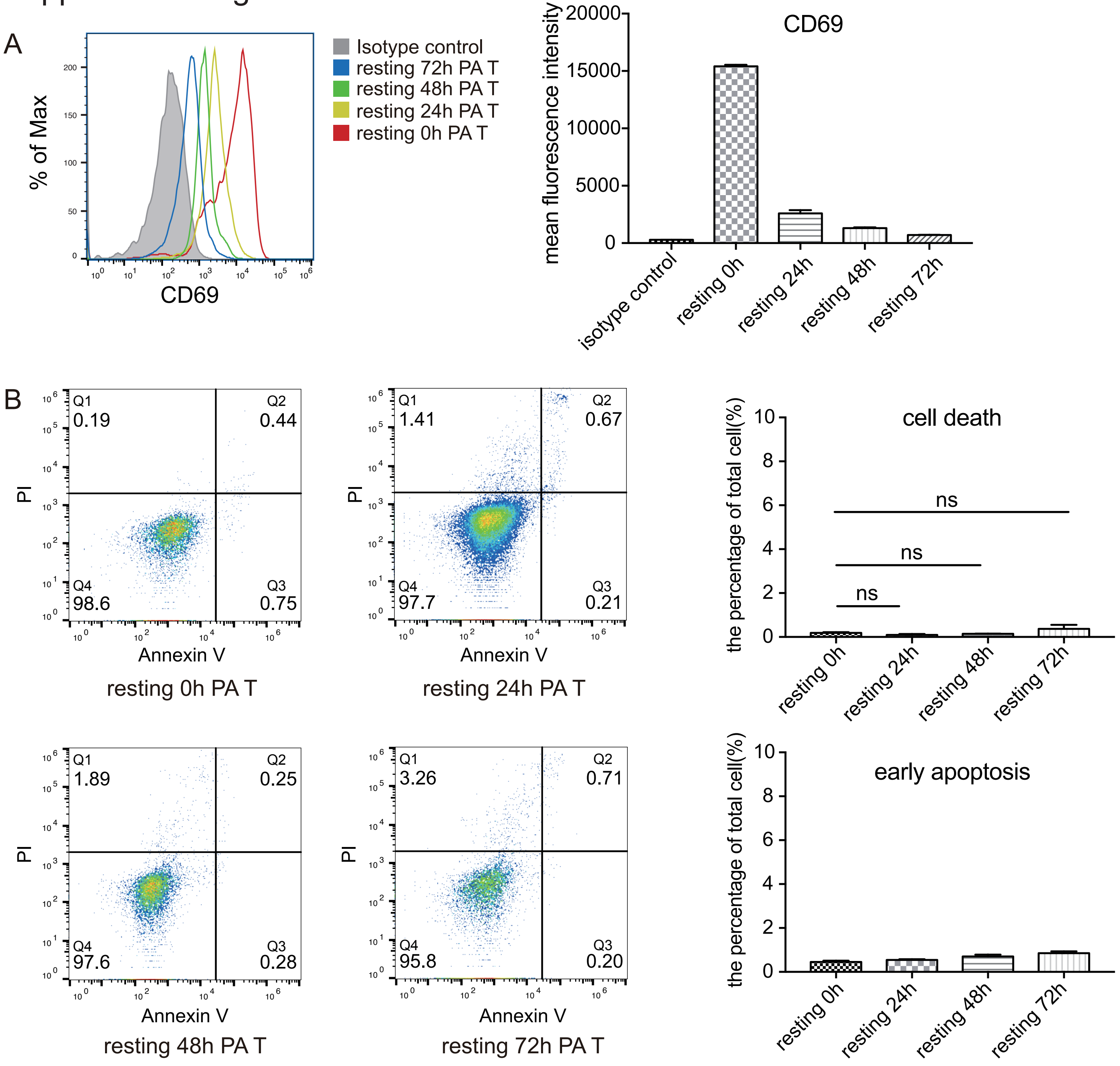

Figure 2: CD69 expression and cell death analyzed during resting different times
PA T cells were harvested by FACS sorting, and rested in fresh 10% FBS media. (A) the peak of CD69 expression and the MFI of CD69 after different resting times. Three replicates in each group (n=3), results are representative of three independent experiments (N=3).
(B) the percentage of cell death and early apoptosis at the different time points into resting by PI/ annexin V staining. Three replicates in each group (n=3), results are representative of three independent experiments (N=3).

Supplemental Figure 3

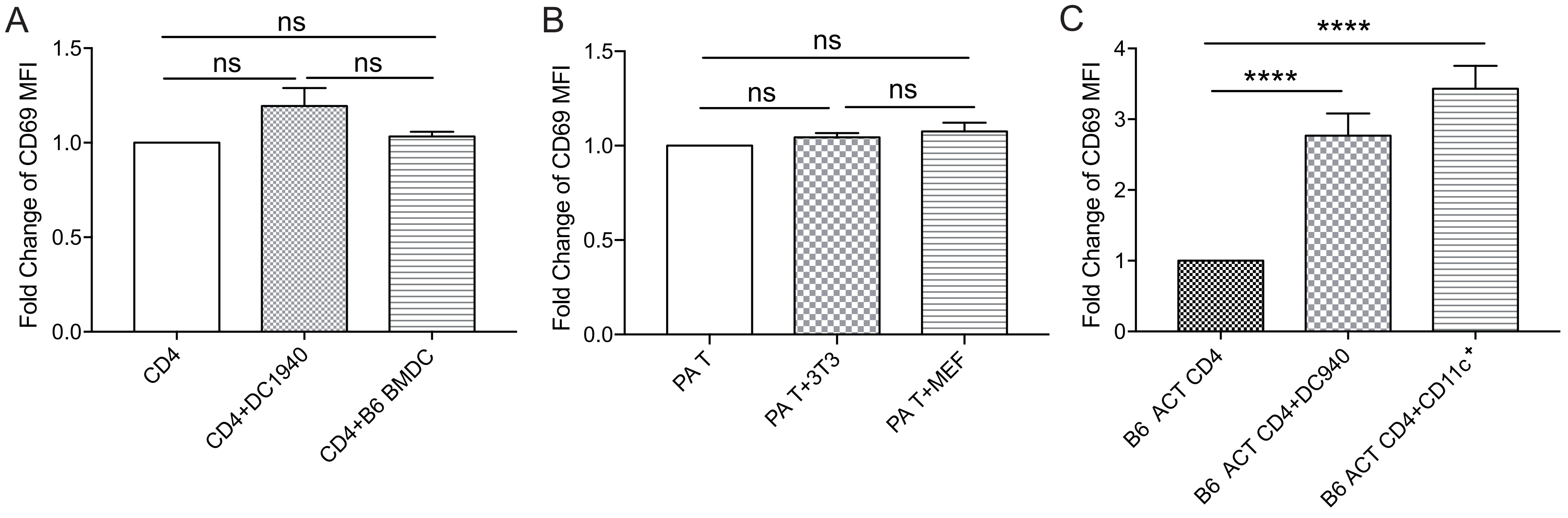

Figure 3. PA T cells upregulate CD69 in DC co-culture
(A) Naïve freshly magnetically isolated OT-II splenic CD4 cells, CD69 expression was compared with those co-cultured with DC1940 cell-line or B6 BMDCs. Pooled all data from three independent experiments are shown. Normalized CD69 mean fluorescence intensity (MFI) by the naïve CD4 T group in multiple independently repeated experiments (N=3) was analyzed for fold change of CD69 MFI. (B) Representative staining of PA T after 48h resting, CD69 expression was compared with those co-cultured with B6 MEF cell-line or 3T3. Pooled all data for fold change of CD69 MFI from four independent experiments are shown. (C) Magnetically isolated naïve CD4 T cells from B6 mice were activated in vitro with anti-CD3e and anti-CD28. Same experiment as in B was performed using B6 splenic CD11c+ cells and DC1940 as the stimulator. Pooled all data for fold change of CD69 MFI from three independent experiments are shown. ns means no significant difference, ****$p<0.0001$ (Unpaired Student’s t test).

Supplemental Figure 4

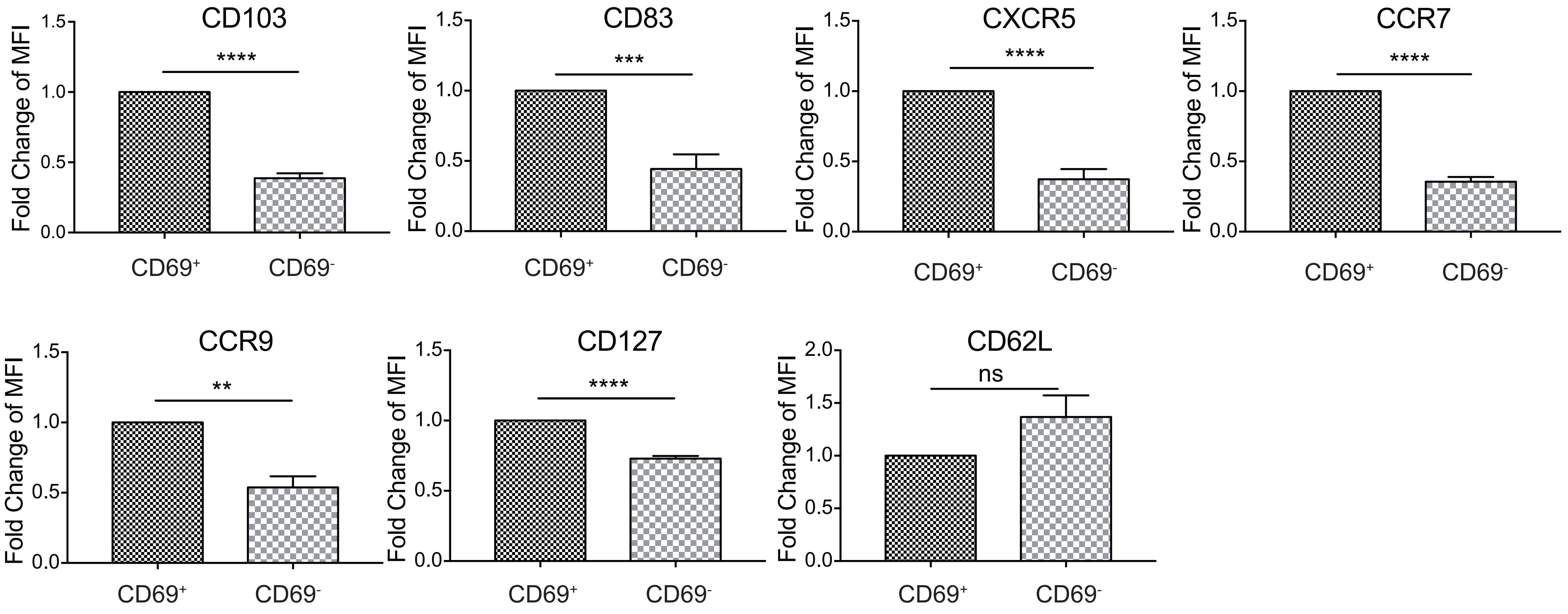

Figure 4. surface marker between CD69+ and CD69-
PA T cells were co-cultured with DC1940 for 24 hours, then CD4+ populations were gated into CD69+ and CD69- cells by FACS. The expression of CD103, CXCR5, CD62L, CCR9, CCR7, CD127 and CD83 between CD69+ and CD69- cells were compared. Normalized the CD103, CXCR5, CD62L, CCR9, CCR7, CD127 and CD83 mean fluorescence intension (MFI) expression by the CD69+ group was analyzed for the fold change of MFI over CD69- group. Results are representative of three independent experiments (N=3). **p<0.01, ***p<0.001, ****p<0.0001, ns stands no significant difference (Unpaired Student's t test).

Supplemental Figure 5

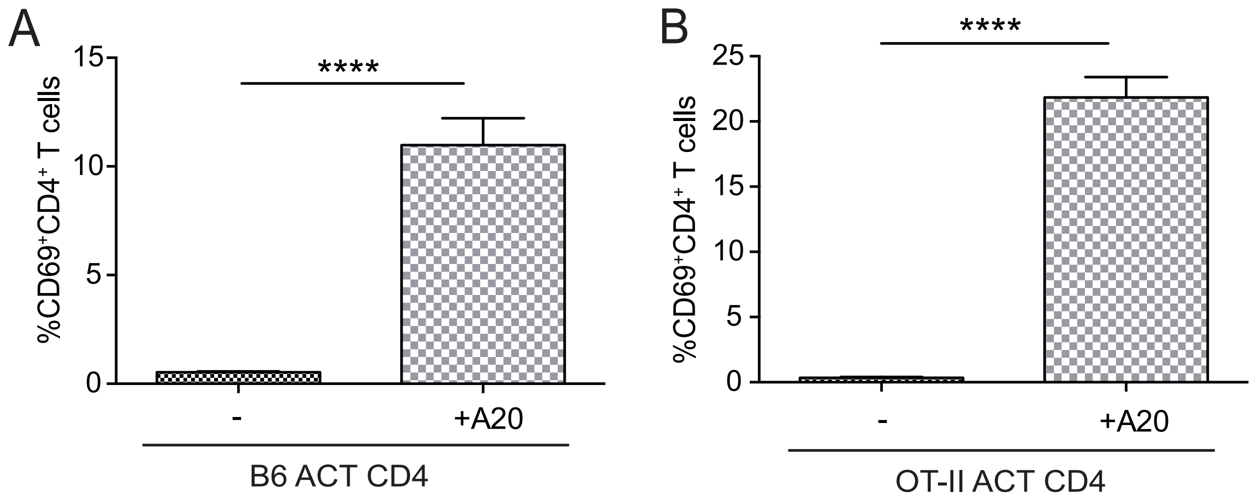

Figure 5. non-specific activation CD4 T cells upregulated CD69 expression in A20 co-culture
(A) Magnetically isolated naïve CD4 T cells from B6 mice were activated in vitro with anti-CD3e and anti-CD28. Then the non-specifically activated CD4 T cells were co-cultured with A20 for 24 hours, and analyzed for their CD69 expression of activated CD4 T cells. ****$p<0.0001$ (Unpaired Student's t test). Three replicates in each group (n=3). Results are representative of three independent experiments (N=3). (B) Magnetically isolated naïve CD4 T cells from OT-II mice were activated in vitro with anti-CD3e and anti-CD28. Same assay as in A was performed. Three replicates in each group (n=3). Results are representative of three independent experiments (N=3). ****$p<0.0001$ (Unpaired Student's t test).

Supplemental Figure 6

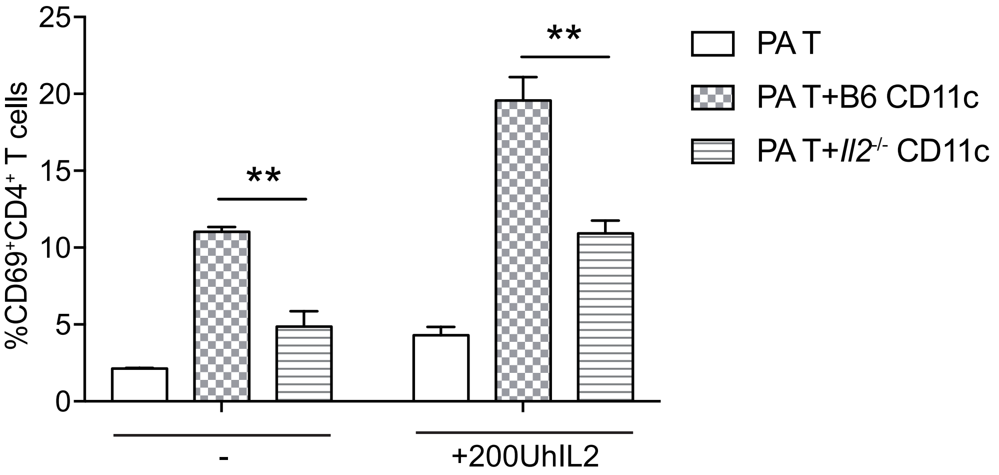

Figure 6. high dose hIL2 can rescue endogenous IL-2 from DCs to PA T
CD69 expression on PA T cells co-cultured with WT and Il2-/- splenic CD11c+ cells, in the presence or absence of added 200U/ml hIL2.
**p<0.01(Unpaired Student's t test). Three replicates in each group (n=3). Results are representative of three independent experiments (N=3).
